# Chinese Glioma Genome Atlas (CGGA): A Comprehensive Resource with Functional Genomic Data from Chinese Glioma Patients

**DOI:** 10.1016/j.gpb.2020.10.005

**Published:** 2021-03-02

**Authors:** Zheng Zhao, Ke-Nan Zhang, Qiangwei Wang, Guanzhang Li, Fan Zeng, Ying Zhang, Fan Wu, Ruichao Chai, Zheng Wang, Chuanbao Zhang, Wei Zhang, Zhaoshi Bao, Tao Jiang

**Affiliations:** 1 Beijing Neurosurgical Institute, Capital Medical University, Beijing 100070, China; 2 Department of Neurosurgery, The Second Affiliated Hospital of Zhejiang University School of Medicine, Hangzhou 310009, China; 3 Department of Neurosurgery, Beijing Tiantan Hospital, Capital Medical University, Beijing 100070, China; 4 Center of Brain Tumor, Beijing Institute for Brain Disorders, Beijing 100069, China; 5 China National Clinical Research Center for Neurological Diseases, Beijing 100070, China

**Keywords:** Glioma, Functional genomics, Chinese Glioma Genome Atlas, Chinese cohort, Database

## Abstract

**Gliomas** are the most common and malignant intracranial tumors in adults. Recent studies have revealed the significance of **functional genomics** for glioma pathophysiological studies and treatments. However, access to comprehensive genomic data and analytical platforms is often limited. Here, we developed the **Chinese Glioma Genome Atlas** (CGGA), a user-friendly data portal for the storage and interactive exploration of cross-omics data, including nearly 2000 primary and recurrent glioma samples from **Chinese cohort**. Currently, open access is provided to whole-exome sequencing data (286 samples), mRNA sequencing (1018 samples) and microarray data (301 samples), DNA methylation microarray data (159 samples), and microRNA microarray data (198 samples), and to detailed clinical information (age, gender, chemoradiotherapy status, WHO grade, histological type, critical molecular pathological information, and survival data). In addition, we have developed several tools for users to analyze the mutation profiles, mRNA/microRNA expression, and DNA methylation profiles, and to perform survival and gene correlation analyses of specific glioma subtypes. This **database** removes the barriers for researchers, providing rapid and convenient access to high‐quality functional genomic data resources for biological studies and clinical applications. CGGA is available at http://www.cgga.org.cn.

## Introduction

Gliomas are the most common intracranial malignant tumors in adults. According to a multi-center cross-sectional study on brain tumors in China, the age-standardized prevalence of primary brain tumors is approximately 22.52 per 100,000 for all populations, with gliomas accounting for 31.1% [1], [2], [3]. Despite advances in current treatment strategies, the survival rate of patients with glioma has not been obviously improved in decades, especially for aggressive gliomas (associated with a poor median survival time of 14.4 months) [4], [5]. According to the 2016 World Health Organization (WHO) classification of central nervous system (CNS) tumors, gliomas are classified from grade II to grade IV by not only histological characteristics but also several molecular pathological features, *e.g.*, *IDH* (*IDH1* and *IDH2*) mutation and chromosome 1p/19q co‐deletion status [6]. Clinically, most lower-grade gliomas (LGGs) progress to glioblastoma (grade IV, GBM) in less than 10 years [6], [7], [8]. Glioma recurrence or malignant progression occurs likely for several reasons: (1) infiltrative tumor cells cannot be completely removed by neurosurgical resection [9], [10]; (2) residual tumor cells cannot be effectively suppressed by limited postoperative treatment options [3], [11], [12]; (3) multiple lesions may progress sequentially [13], [14]; (4) tumor cell cloning occurs rapidly under chemotherapy and/or radiotherapy [7], [15]; and (5) tumor cells readily adapt to the immunosuppressive tumor microenvironment [16], [17]. Glioma research is greatly hindered by limited data resources. Therefore, it is essential to collect clinical specimens and provide genomic sequencing data to the glioma research community.

Recently, high‐throughput technologies have been extended to characterize genomic status including but not limited to DNA methylation modification, genetic alteration, and gene expression regulation. In the cancer research community, major large-scale projects, such as The Cancer Genome Atlas (TCGA, which includes 516 LGG samples and 617 GBM samples as of October 18, 2019) [18] and the International Cancer Genome Consortium [ICGC, which includes 80 adult GBM samples and 50 pediatric GBM samples (excluding the TCGA samples) as of April 3, 2019] [19], [20], have generated an unparalleled amount of functional genomic data. These projects have changed our understandings of cancers and led to breakthroughs in diagnosis, treatments, and prevention. Importantly, they have provided opportunities for discovery and validation to researchers worldwide. However, the data generated by these projects are often difficult to access, analyze, and visualize, especially for researchers with little bioinformatics skill. These limitations have greatly hindered the use of functional genomics data to obtain novel findings of significance for drug development and clinical treatments. Although several webservers, *e.g.*, cBioportal [21], [22] and GlioVis [23], have been constructed to analyze multi‐dimensional glioma data, they ignore the heterogeneity in tumors, as data obtained from recurrent glioma samples and subtype analyses are lacking.

Here, we introduce the Chinese Glioma Genome Atlas (CGGA, http://www.cgga.org.cn), an open‐access and easy-to‐use platform for the interactive exploration of multi-dimensional functional genomic datasets collected from nearly 2000 glioma samples from Chinese cohorts. The database currently contains a wide range of data derived from whole-exome sequencing (WES, 286 samples), mRNA sequencing (1018 samples) and microarray (301 samples), DNA methylation microarray (159 samples), and microRNA microarray analyses (198 samples), as well as comprehensive clinical data. Furthermore, we developed various online tools to browse mutational landscape profiles, mRNA/microRNA expression profiles, and DNA methylation profiles, and to perform survival and correlation analyses of specific subtypes. We hope that CGGA removes the barriers for researchers who need fast and convenient access to high‐quality functional genomic data resources.

## Database implementation

**Figure 1:**
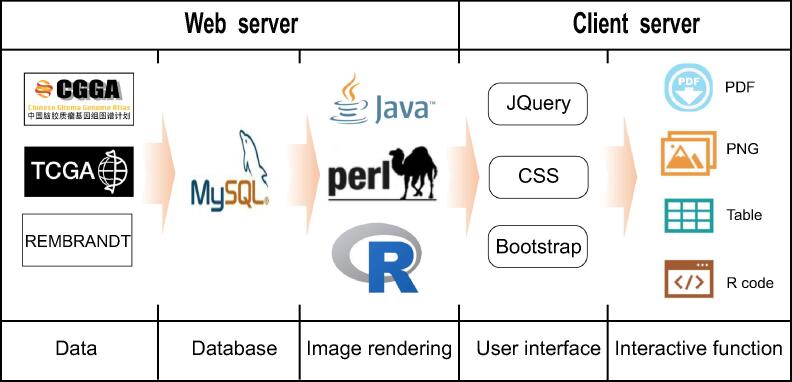
**Schematic of CGGA illustrating thedata processing and display approaches**

In CGGA, all data were organized using MySQL 14.14 based on relational schema, which will be supported in future CGGA updates. The website code was written based on Java Server Pages using the Java Servlet framework. The website is deployed on the Tomcat 6.0.44 web server and runs on a CentOS 5.5 Linux system. JQuery was used to generate, render, and manipulate data for visualization. The ‘Analyze’ module was realized by Perl and R script. The CGGA website has been fully tested in Google Chrome and Safari browsers. The design of CGGA is displayed in Figure 1.

## Database content and usage

### Database content

The CGGA database is designed to archive functional genomic data and to allow the interactive exploration of multidimensional datasets from both primary and recurrent gliomas in Chinese cohorts. The database is available at http://www.cgga.org.cn. Currently, CGGA contains WES (286 samples), mRNA sequencing (a total of 1018 samples, with batch 1 comprising 693 samples and batch 2 comprising 325 samples), mRNA microarray (301 samples), DNA methylation microarray (159 samples), and microRNA microarray (198 samples) data, and detailed clinical data (including age, gender, chemoradiotherapy status, WHO grade, histological type, critical molecular pathological information, and survival data). Detailed statistical information of each dataset is provided in Table 1. Out-house sequencing data from TCGA (702 samples) and the Repository of Molecular Brain Neoplasia Data (REMBRANDT, 475 samples) can be acquired on the download page. We have organized web interface of CGGA according to the four main functional features: (i) Home, (ii) Analyze, (iii) Tools, and (iv) Download. In what follows, we provide an example illustrating how to use CGGA.

**Table 1:** **Clinical and phenotypical characteristics of datasetsin CGGA database**

### The homepage

On the ‘Home’ page, CGGA provides a statistical table of all collected datasets, including dataset name, data type, number of samples in each subgroup, clinical data, and analysis purpose. For instance, we have performed mRNA sequencing on 1018 glioma samples and obtained 693 samples in batch 1 and 325 samples in batch 2 (including 282 primary LGG samples, 161 recurrent LGG samples, 140 primary GBM samples, and 109 recurrent GBM samples in batch 1 and 144 primary LGG samples, 38 recurrent LGG samples, 85 primary GBM samples, 24 recurrent GBM samples, and 30 secondary GBMsamples in batch 2). Of note, CGGA is the first database to archive functional genomic data for both recurrent LGGs and GBM samples. In addition, users can view the results of the analysis of each dataset by clicking on hyperlinks on the ‘Home’ page. The ‘Download’ and ‘Help’ pages can be accessed directly from the ‘Home’ page.

### The analyses and results

**Figure 2:**
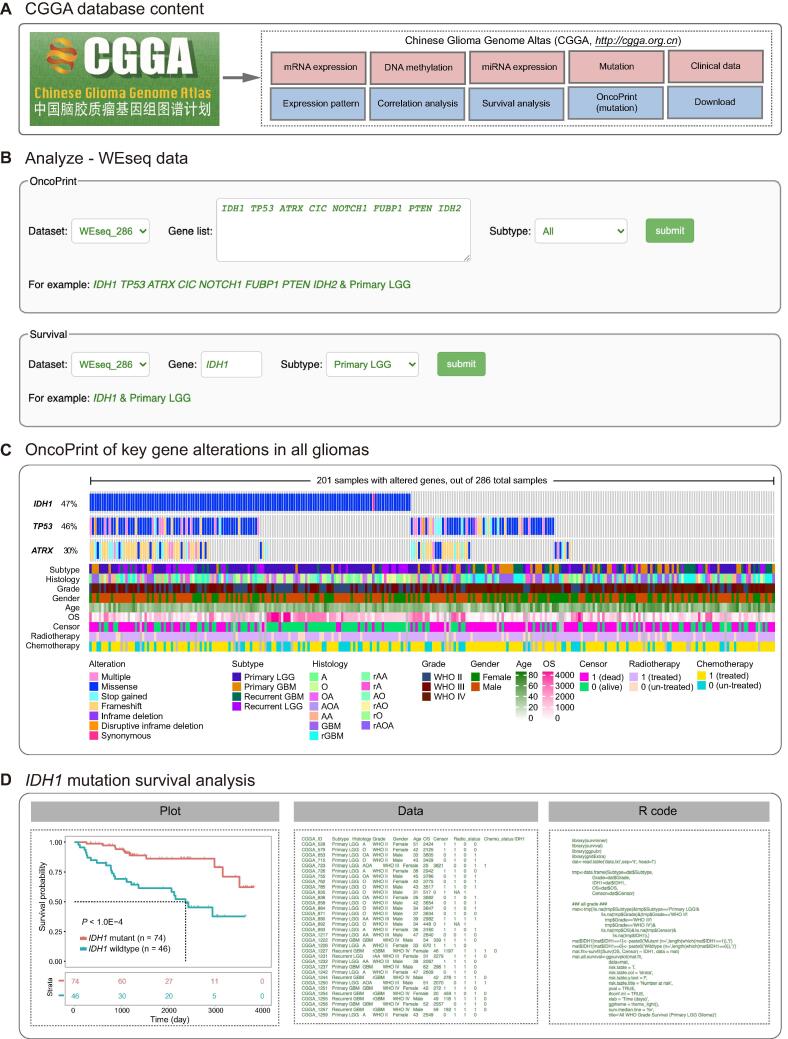
**Main contents of CGGA database and the functionality of WEseq analysis** **A.** CGGA contains whole-exome sequencing, mRNA and microRNA expression, DNA methylation data, clinical data, and several analysis modules. **B.** The web image in the WEseq analysis page to search the OncoPrint and prognostic value of target genes. **C.** The mutation profile in all glioma samples included (in the ‘WEseq_286’ dataset). **D.** Survival analysis of primary LGG patients with *IDH1* mutation. Left: the plot for overall survival of primary LGG patients with wildtype or mutant *IDH1* (in the ‘WEseq_286’ dataset); middle: the data used to generate the plot; right: the R code used to generate the plot. LGG, lower-grade glioma; GBM, glioblastoma; A, astrocytoma; O, oligodendroglioma; OA, oligo‐astrocytoma; AOA, anaplastic oligo-astrocytoma; AA: anaplastic astrocytoma; rGBM: recurrent glioblastoma; rAA, recurrent anaplastic astrocytoma; rA, recurrent astrocytoma; AO, anaplastic oligodendroglioma; rAO, recurrent anaplastic oligodendroglioma; rO, recurrent oligodendroglioma; rAOA, recurrent anaplastic oligo‐astrocytoma.

To facilitate analysis of the CGGA data, especially for bioinformatics beginners, we developed four online modules in the ‘Analyze’ tab (Figure 2). ‘WEseq data’, ‘mRNA data’, ‘methylation data’, and ‘microRNA data’ are included for analyzing the WES, mRNA expression, DNA methylation, and microRNA expression data, respectively (Figure 2A). A key feature of CGGA is its ease of use. In the example below, we illustrate the usage of the ‘Analyze’ tab in CGGA.

On the ‘WEseq data’ page, users can visualize the mutational profile of a gene set of interest and perform a survival analysis of a specific gene of interest in specific glioma subtypes (Figure 2B). In the ‘OncoPrint’ section, users are guided to (a) input a gene set of interest, for example, *IDH1*, *TP53*, and *ATRX*; and (b) select a subtype of interest, for example, ‘All’. Based on user input, the tool automatically generates results and displays to the users. In the results, data for each case or patient are presented in columns, each row corresponds to a gene; different kinds of mutations are marked in colors and a heatmap is presented below the table depicting clinical information (Figure 2C). The ‘OncoPrint’ section can be very useful for visualizing the mutational profile of a gene set of interest in a specific glioma subtype and intuitively revealing mutual exclusivity or cooccurrence for a gene pair. In the example above, the mutations in gene *IDH1* (47%), *TP53* (46%), and *ATRX* (30%) were the most common mutations in all glioma samples included. In the ‘Survival’ section, users can input a specific gene (*e.g.*, *IDH1*) and select a subtype (*e.g.*, ‘Primary LGG’) to investigate the association of gene mutation with survival. Consistent with previous studies [24], primary LGG patients with *IDH1* mutation show better overall survival than patients carrying wildtype *IDH1* (*P* < 0.0001, Figure 2D, left). The results from the ‘WEseq data’ section can be exported in PDF format. To ensure repeatability, the input data (Figure 2D, middle) and R code (Figure 2D, right) are provided, enabling users to reproduce the figure with customized options according to their own need.

**Figure 3:**
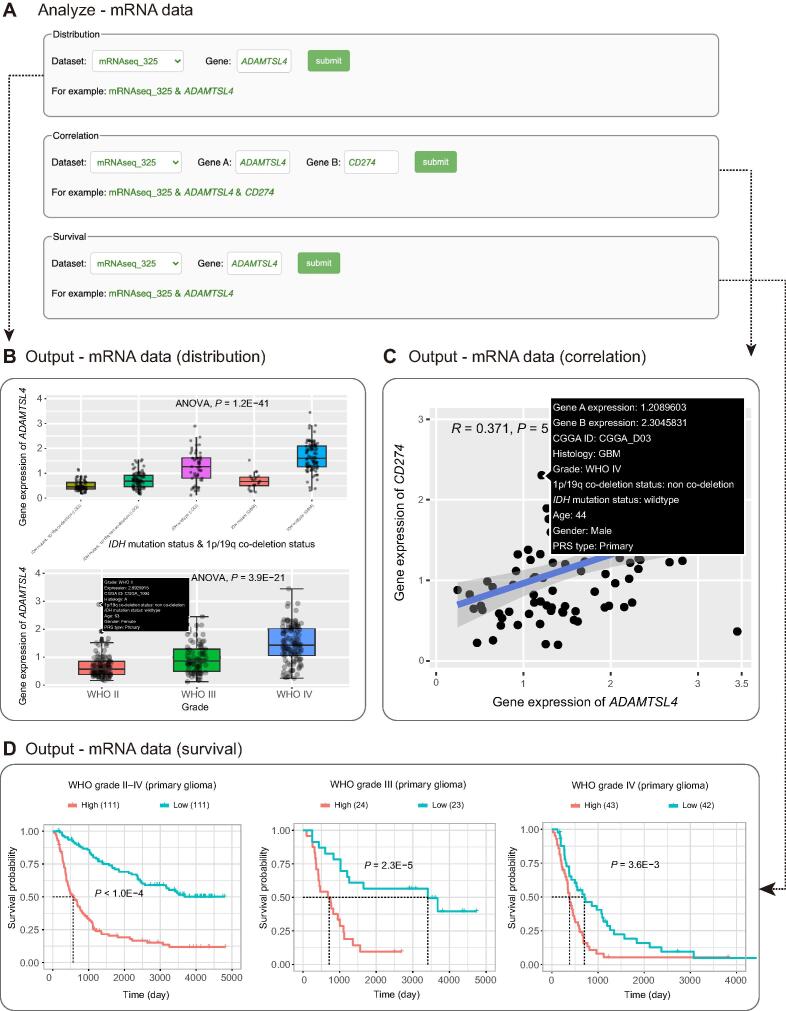
**Examples of CGGA RNA-seq analysis** **A.** The screenshot of the RNA-seq analysis page to search the distribution, correlated genes, and prognostic value of target genes. **B.** The *ADAMTSL4* gene expression distribution in primary gliomas based on the 2016 WHO grading system (in the ‘mRNAseq_325’ dataset). **C.** The correlation of gene expression between *ADAMTSL4* and *CD274* (in the ‘mRNAseq_325’ dataset). **D.** The overall survival of glioma patients with low and high expression of *ADAMTSL4* (in the ‘mRNAseq_325’ dataset).

On the ‘mRNA data’ page, users can perform the distribution of gene expression, correlation, and survival analyses for a specific gene in a specific glioma subtype (Figure 3A). Three mRNA datasets are available to users, including two batches of RNA-seq datasets (batch 1: 693 samples; batch 2: 325 samples) and one microarray dataset (301 samples). In the ‘Distribution’ section, users can display one gene distribution pattern for each glioma subtype by selecting a dataset (*e.g.*, ‘mRNAseq_325’) and inputting a gene name of interest (*e.g.*, *ADAMTSL4*).

Upon hovering the mouse over each point, the expression level and clinical information of each case appear in a pop-up window. The results show the gene expression pattern in each glioma subtype classified based on clinical information. In our illustrative case, similar to our previous studies [25], gene *ADAMTSL4* was shown to be differentially expressed according to the WHO 2016 classification based on the *IDH* mutation and/or 1p/19q co‐deletion status and WHO grade (Figure 3B). In addition, a unique feature of the CGGA dataset is the inclusion of recurrent gliomas. This module allows users to infer whether a gene may be a candidate factor that drives malignant progression if it is differentially expressed between primary and recurrent gliomas. In the ‘Correlation’ section, the user can examine the co‐expression pattern by selecting a dataset (*e.g.*, ‘mRNAseq_325’) and entering a gene pair (*e.g.*, *ADAMTSL4* and *CD274*). As a result, the co-expression patterns in each glioma subtype are displayed with the results of Pearson’s correlation and the *P* value (Figure 3C). In the ‘Survival’ section, users can perform survival analysis based on gene expression by selecting a dataset (*e.g.*, ‘mRNAseq_325’) and inputting a gene (*e.g.*, *ADAMTSL4*). In our illustrative case, all primary glioma patients with low *ADAMTSL4* expression have better overall survival than those with high *ADAMTSL4* expression (*P* < 0.0001, Figure 3D left; *P* = 0.00023, Figure 3D middle; *P* = 0.0036, Figure 3D right). The results above from the ‘mRNA data’ section are consistent with the results of our previous study [25]. Similar to the ‘mRNA data’ page, on ‘methylation data’ page and the ‘microRNA data’ page, users can view the methylation/miRNA distribution and perform correlation and survival analyses.

Further analyses can be accomplished in the ‘Tools’ section, such as differential expression analysis, clustering analysis, and correlation analysis. An expression matrix can be downloaded and rearranged by the user, and the user can upload an input matrix following the instructions. The resulting graph can be downloaded in PDF format.

### Data acquisition

Users can download all datasets on the ‘Download’ page. Each data type is saved at the gene and/or probe level and is then combined with available clinical data, including basic clinical information, survival, and therapy information. The raw sequencing data can be accessed at the National Genomics Data Center (NGDC, https://ngdc.cncb.ac.cn) by filing an application online.

## Method

### Clinical specimen collection

Glioma tissues and corresponding genomic data and patient follow-up information were obtained from Beijing Tiantan Hospital at Capital Medical University, Tianjin Medical University General Hospital, Sanbo Brain Hospital at Capital Medical University, the Second Affiliated Hospital of Harbin Medical University, the First Affiliated Hospital of Nanjing Medical University, and the First Hospital of China Medical University. According to the pathological reassessment of independent neuropathologists, all the subjects were consistently diagnosed with glioma and were then further classified according to the 2007/2016 WHO classification system. The specimens were collected according to protocols approved by the Institutional Review Boards of Beijing Tiantan Hospital (Approval No. IRB KY2013-017-01) and frozen in liquid nitrogen within 5 min of resection.

### Data processing for WES data

Genomic DNA from each tumor and the matched blood sample was extracted and assessed for integrity by 1% agarose gel electrophoresis. The DNA was subsequently fragmented and subjected to quality control, and then pair-end libraries were prepared. The Agilent SureSelect kit v5.4 (Cat No. 5990-9857, Santa Clara, CA) was used for target capture. Sequencing was performed on a HiSeq 4000 platform (Illumina, San Diego, CA) using pair-end sequencing strategy. Valid DNA sequencing data were mapped to the reference human genome (UCSC hg19) using Burrows-Wheeler Aligner (v0.7.12-r1039, bwa mem) [26] with default parameters. Then, SAMtools (V1.2) [27] and Picard (V2.0.1, Broad Institute, Cambridge, MA) were used to sort the reads by coordinates and mark duplicates. Statistics such as sequencing depth and coverage were calculated based on the resultant BAM files. SAVI2 was used to identify somatic mutations (including single nucleotide variations and short insertion/deletions) as previously described [7], [8]. Briefly, in this pipeline, SAMtools mpileup and bcftools (V0.1.19) [28] were used to perform variant calling; then, the preliminary variant list was filtered to remove positions with insufficient sequencing depth, positions with only low-quality reads, and positions that were biased toward either strand. Somatic mutations were identified and evaluated by an empirical Bayesian method. In particular, mutations with a mutation allele frequency in tumors significantly higher (*P* < 0.05) than that in normal controls were selected.

### Data processing for mRNA sequencing data

Prior to library preparation, total RNA was isolated using the RNeasy Mini Kit (Cat No. 74104, Qiagen, Dusseldorf, Germany) according to the manufacturer’s instructions. Pestle and QIAshredder (Cat No. 79654, Qiagen) were used to disrupt and homogenize frozen tissue. RNA intensity was evaluated using Agilent 2100 Bioanalyzer, and only high‐quality samples with an RNA Integrity Number (RIN) value greater than or equal to 6.8 were used to construct the sequencing library. Typically, 1 μg of total RNA was used with the TruSeq RNA library preparation kit (Cat No. RS-122-2001, Illumina) in accordance with low-throughput protocols, except for the use of SuperScript III reverse transcriptase (Cat No.18080044, Invitrogen, Carlsbad, CA) to synthesize first-strand cDNA. After PCR enrichment and purification of adapter-ligated fragments, the concentration of DNA with adapters was determined with 7500 Fast Real-Time PCR Systems (Applied Biosystems, Carlsbad, CA) using primers QP1 5′-AATGATACGGCGACCACCGA-3′ and QP2 5′-CAAGCAGAAGACGGCATACGAGA-3′. The length of the DNA fragment was measured using an Agilent, 2100 Bioanalyzer with a median insert size of 200 nucleotides. Then, RNA-seq libraries were sequenced using the Illumina HiSeq 2000, 2500, or 4000 Sequencing System. The libraries were prepared using the paired-end strategy with a read length of 101 bp, 125 bp, or 150 bp. Base-calling was performed by the Illumina CASAVA V1.8.2 pipeline. RNA-seq mapping and quantification were performed by STAR (V2.5.2b) [29] and RSEM (V1.2.31) software [30]. Briefly, the reads were aligned to the human genome reference (GENCODE v19, hg19) with STAR, and then sequencing read counts for each GENCODE gene were calculated using RSEM. The expression levels of different samples were merged into a fragments per kilobase transcriptome per million fragments (FPKM) matrix. We defined the expressed gene only if FPKM is larger than 0 in half of the samples. We retained only the expressed genes in the mRNA expression profile.

### Data processing for mRNA microarray data

A rapid hematoxylin & eosin stain for frozen sections was applied to each sample to assess the tumor cell proportion before RNA extraction. RNA was extracted only from the samples with > 80% tumor cells. Total RNA was extracted from frozen tumor tissues with the Ambion mirVana miRNA Isolation kit (Cat No. AM1560, Austin, TX) as described previously [31]. The ND-1000 spectrophotometer (NanoDrop, Wilmington, DE) was applied to evaluate the quality and concentration of the extracted total RNA, and the Agilent 2100 Bioanalyzer was used to assess RNA integrity. Then, the qualified RNA was collected for further procedures. cDNA and biotinylated cRNA were synthesized and hybridized to the Agilent Whole Human Genome Array according to the manufacturer's instructions. Finally, the array-generated data were analyzed by the Agilent G2565BA Microarray Scanner System and Agilent Feature Extraction software (V9.1). GeneSpring GX11.0 was applied to calculate probe intensity.

### Data processing for methylation microarray data

A hematoxylin & eosin-stained frozen section was prepared for assessment of the percentage of tumor cells before RNA extraction. Only samples with > 80% tumor cells were selected. Genomic DNA was isolated from frozen tumor tissues using the QIAamp DNA Mini Kit (Cat No. 51304, QIAGEN) according to the manufacturer’s protocol. DNA concentration and quality were measured using the NanoDrop ND-1000 spectrophotometer. We used the Illumina Infinium HumanMethylation27 Bead-Chip. The Bead-Chip contains 27,578 highly informative CpG sites covering more than 14,000 human RefSeq genes. This array allows researchers to interrogate all of these sites per sample at a single nucleotide resolution. Bisulfite modification of DNA, chip processing, and data analysis were performed following the manufacturer’s manual at the Wellcome Trust Centre for Human Genetics Genomics Lab, Oxford, UK. The array results were analyzed using the BeadStudio software (Illumina).

### Data processing for microRNA microarray data

Total RNA was extracted from frozen tissues by using the Ambion mirVana miRNA Isolation Kit, and its concentration and quality were determined with the NanoDrop ND-1000 spectrophotometer. microRNA expression profiling was performed using the Illumina human v2.0 miRNA Expression BeadChip with 1146 microRNAs covering 97% of the miRBase 12.0 database according to the manufacturer’s instructions.

## Discussion and perspectives

The current version of CGGA is the first release of this database, which includes multi‐dimensional functional genomic glioma data, *e.g.*, WES, mRNA, and microRNA expression, and DNA methylation data, for nearly 2000 samples from Chinese cohorts. Considering the significance of these data for glioma research, we have decided to make CGGA publicly available for worldwide researchers. To the best of our knowledge, CGGA is the first database archiving functional genomic data of both recurrent LGG samples and GBM samples. In addition, CGGA provides online interactive functionalities, including mutational profile, gene expression distribution pattern, correlation, and survival analyses. Phenotype-focused exploration, differential expression analysis, and clustering analysis can be performed by uploading rearranged gene matrixes and online tools. These features will be convenient for obtaining and validating novel findings of biological significance for bioinformatics beginners.

However, the current version of CGGA is still nascent. The visitor-interactive functionalities will be improved in future updates. Unlike TCGA, there are no neuroimaging data in CGGA currently, which is a limitation of the database. Such data will be uploaded in the near future. In addition to addressing these shortcomings, future improvement of our CGGA database is planned. First, relying on the Beijing Neurosurgical Institute, Beijing Tiantan Hospital and Chinese Glioma Cooperative Group (CGCG) Research Network, we will continue to collect glioma tissue samples, perform cross-omics sequencing/ microarray analyses, and update the database regularly. In addition, we plan to provide single-cell sequencing data that match a subset of patients in the existing cohort. Furthermore, we will improve the integrity of the molecular pathological data by retrospectively checking medical records or reanalyzing pathological slices.

In summary, CGGA provides access to multi-omics sequencing data on Chinese cohorts for the global research community. It provides a user-friendly interface for obtaining integrated datasets, performing intuitive visualized analysis, and downloading these datasets. CGGA greatly reduces the barriers for glioma researchers to gain access to complex functional genomic data, allowing them to harness functional genomic data for important biological insights and identify potential clinical applications.

## Ethical statement

All studies performed were approved by the Institutional Review Board (IRB) of Beijing Tiantan Hospital, Capital Medical University, and were conducted according to the principles of the Helsinki Declaration. Written informed consent were obtained from all patients.

## Data availability

All data referred to in this article can be found online at http://www.cgga.org.cn. The raw sequence data reported in this article have been deposited in the Genome Sequence Archive [32] at the National Genomics Data Center, Beijing Institute of Genomics, Chinese Academy of Sciences / China National Center for Bioinformation (GSA: HRA000071, HRA000073, and HRA000074), and are publicly accessible at http://bigd.big.ac.cn/gsa-human.

## CRediT authorship statement

**Zheng Zhao:** Methodology, Software, Writing - original draft, Visualization. **Ke-Nan Zhang:** Methodology, Formal analysis, Investigation, Writing - review & editing. **Qiangwei Wang:** Formal analysis, Investigation, Data curation, Writing - review & editing. **Guanzhang Li:** Investigation, Data curation. **Fan Zeng:** Investigation, Data curation. **Ying Zhang:** Data curation. **Fan Wu:** Resources, Data curation. **Ruichao Chai:** Resources, Data curation. **Zheng Wang:** Resources, Data curation. **Chuanbao Zhang:** Data curation. **Wei Zhang:** Conceptualization, Validation, Project administration, Funding acquisition. **Zhaoshi Bao:** Conceptualization, Supervision. **Tao Jiang:** Conceptualization, Resources, Supervision, Project administration, Funding acquisition. All authors read and approved the final manuscript.
